# Could ceftriaxone be a viable alternative to penicillin for the treatment of ocular syphilis?

**DOI:** 10.1128/aac.00080-24

**Published:** 2024-05-06

**Authors:** Xin Gu, Haikong Lu, Yilan Yang, Lin Zhu, Mei Shi, Zhifang Guan, Liyan Ni, Ruirui Peng, Wei Zhao, Juan Wu, Tengfei Qi, Pingyu Zhou

**Affiliations:** 1 Institute of Sexually Transmitted Disease, Shanghai Skin Disease Hospital, School of Medicine, Tongji University, Shanghai, China; University of Fribourg, Fribourg, Switzerland

**Keywords:** ceftriaxone, penicillin, ocular syphilis, treatment

## Abstract

This study was conducted to compare the effectiveness of ceftriaxone with that of aqueous crystalline penicillin G in treating ocular syphilis. We conducted a retrospective study from 2010 to 2021. Syphilis patients were administered either ceftriaxone (2 g intravenously daily for 14 days) or aqueous crystalline penicillin G [4 million units (MU) intravenously every 4 h for 14 days] as therapeutic interventions. Subsequently, we utilized these two groups to assess the serological results, cerebrospinal fluid analysis, and visual acuity at time intervals spanning 3 to 6 months post-treatment. A total of 205 patients were included, with 34 assigned to the ceftriaxone group and 171 to the penicillin group. The median age of patients was 56 years, with an interquartile range of 49–62 years, and 137 of them (66.8%) were male. Between 3 and 6 months after treatment, 13 patients (38.2%) in the ceftriaxone group and 82 patients (48.0%) in the penicillin group demonstrated effective treatment based on the clinical and laboratory parameters. The crude odds ratio (OR) was 0.672 (95% confidence interval [CI]: 0.316–1.428, *P* = 0.301), indicating no significant difference in effectiveness between the two groups. Thirty patients (17.5%) in the penicillin group and six patients (17.6%) in the ceftriaxone group did not experience successful outcomes. Notably, no serious adverse effects were reported in both the groups. There was no significant difference in the effectiveness of ceftriaxone and aqueous crystalline penicillin G in treating ocular syphilis. The administration of ceftriaxone without requiring hospitalization presents a convenient and safe alternative treatment option for ocular syphilis.

## INTRODUCTION

Syphilis is a sexually transmitted infectious disease caused by *Treponema pallidum* and has spread across the globe (1–3). In 2021, the number of reported cases of syphilis in China was 480,020, marking the highest count among all countries worldwide and signifying a 3.4% increase compared to the previous year (4). This places syphilis as the third-most prevalent infectious disease in China, based on the incidence rates (4). While the incidence of syphilis has witnessed a significant upsurge in China in recent years, the China Centers for Disease Control and Prevention (CCDC) does not routinely collect national surveillance data on ocular syphilis. Consequently, the actual prevalence of ocular syphilis in China remains undisclosed. In other countries facing a resurgence of syphilis, there have been notable escalations in cases of ocular syphilis as well (5). In the United States, for instance, within the entirety of syphilis surveillance cases, 0.53% and 0.65% of individuals showed ocular symptoms in the years 2014 and 2015, respectively (6).

Ocular involvement can occur at any stage of syphilis (7), with uveitis being the most frequently reported presentation (8–11). Given the absence of a vaccine, early diagnosis and timely intervention are critical in ocular syphilis cases to preserve vision recovery. Delayed treatment of ocular syphilis can lead to unfavorable outcomes, including irreversible blindness (12). It is noteworthy that ocular syphilis is regarded on par with neurosyphilis (NS) and therefore should be managed following NS recommendations, even in cases where cerebrospinal fluid (CSF) results appear normal (13). The gold-standard treatment recommendation for NS is a 10–14-day course of intravenous aqueous crystalline penicillin G, with dosages ranging from 18 to 24 million units (MU) daily, administered in increments of 3 to 4 million units every 4 h (13). However, the requirement of a 2-week hospitalization for treatment often proves impractical for patients with ocular syphilis, particularly among younger individuals experiencing only mild visual impairment, thereby leading to treatment delays.

In cases where a patient is allergic to penicillin, then CDC guidelines for sexually transmitted infections recommend the utilization of desensitization therapy (13). This procedure involves subjecting the patient to penicillin under controlled conditions, and it necessitates execution within an intensive care unit (ICU) setting. It is important to note that even if desensitization proves successful, subsequent penicillin therapy should also be administered within the confines of an ICU. However, the implementation of penicillin desensitization therapy is not established as a practical practice in China. This specific treatment approach lacks a precedent within the country, rendering its adoption unfeasible.

Ceftriaxone is a broad-spectrum antibiotic that is often used to treat various bacterial infections. It emerged as a potential alternative to penicillin in the late 1980 s and has been employed in treating NS (14, 15). This regimen offers the advantage of once or twice daily administration. Although previous studies have reported instances of successful treatment of ocular syphilis using ceftriaxone, these findings primarily originated from case reports and limited-scale investigations (16–18).

The aim of the study was to ascertain whether ceftriaxone matches the effectiveness of aqueous crystalline penicillin G in treating ocular syphilis. If ceftriaxone indeed proves to be as effective as penicillin, it could potentially widen the array of treatment choices available for patients with ocular syphilis. This might include the option of outpatient treatment, thereby increasing patient compliance with treatment protocols.

## MATERIALS AND METHODS

### Study population

This retrospective cohort study encompassed all patients diagnosed with ocular syphilis at the Shanghai Skin Disease Hospital within the timeframe of 2010 to 2021. The management of all patients involved collaborative efforts from the hospital’s multidisciplinary syphilis team, which included specialists in neurology, ophthalmology, and dermatology. Eligibility criteria for study inclusion encompassed individuals aged 18 years and above, regardless of gender, who had a confirmed diagnosis of ocular syphilis. Eligibility criteria for study exclusion comprised pregnant or lactating patients, individuals lacking follow-up data, and those not treated with ceftriaxone or penicillin.

The study was approved by the Shanghai Skin Disease Hospital Ethics Committee (reference number: 2020-16). Written and informed consent was obtained from all patients.

### Data collection

A comprehensive set of data was retrieved from hospital records for each patient. This encompassed fundamental demographic particulars, such as age and gender, along with detailed information from ocular examination. All patients with ocular syphilis underwent a complete ocular examination, HIV screening, testing for previous HIV status, serological testing for syphilis, and lumbar puncture (LP) for the analysis of CSF. These evaluations were conducted at the time of diagnosis and again within an interval of 3 to 6 months after the initiation of treatment.

### Diagnostic and classification criteria

Diagnosis of ocular syphilis was based on positive results for both non-treponemal and treponemal serological tests, coupled with clinical manifestations demonstrating ocular disease. The diagnosis of NS was made based on reactive CSF-Venereal Disease Research Laboratory (VDRL) and CSF-*Treponema pallidum* particle agglutination (TPPA) tests in the absence of substantial contamination of the CSF with blood (CSF appears light red or red in color). A presumption of NS was made when the CSF-VDRL test yielded non-reactive results, but a reactive CSF-TPPA test with either or both of the following: (i) a CSF protein concentration >450 mg/L; (ii) a CSF white blood cell (WBC) count ≥8 /µL in the absence of other known causes for abnormalities (19, 20).

Ocular manifestations were evaluated by an ophthalmologist and categorized into five primary groups, each corresponding to infections affecting distinct eye structures: uveitis (encompassing anterior uveitis, posterior uveitis, and panuveitis), optic neuritis, optic atrophy, conjunctivitis, and others (patients with cranial neuropathy, ocular palsy, or pupil change) (7).

### Treatment and follow-up

Patients were stratified into two distinct groups based on their treatment regimens: (i) aqueous crystalline penicillin G (4MU intravenously every 4 h for 14 days) and (ii) ceftriaxone sodium (Rocephin) intravenously (2 g daily for 14 days) due to their penicillin allergy (in general, patients were treated with penicillin, but when patients were allergic to penicillin, they were treated with ceftriaxone).

All the patients were regularly monitored for their clinical symptoms and subjected to periodic reviews of serum RPR titer and CSF assessments within the 3–6-month post-treatment period. The investigation team retrospectively evaluated clinical and laboratory responses at this follow-up interval. Serological response criteria were established as either a negative RPR outcome or a ≥ 4 fold reduction in the titer at 3–6 months post-treatment. CSF response criteria were defined as either a ≥ 2 fold decrease in the VDRL titer or a return to normal WBC count if previously elevated. The best-corrected visual acuity was evaluated using a standardized logarithmic visual acuity chart, where a shift of one line indicated an improvement or deterioration in visual acuity. The clinical response was categorized into three classes: Effective: defined as an enhancement in both laboratory indicators (serological and CSF) as well as visual parameters. Improved: defined as an improvement in either laboratory indicators (serological or CSF) or visual parameters. Ineffective: defined as no improvement in either the laboratory indicators or visual parameters.

### Statistical analysis

For continuous variables, medians and interquartile range values were presented, whereas categorical variables are expressed as n (%). Baseline data comparisons between the ceftriaxone and penicillin groups were conducted. All continuous variables were tested for normality, and disparities between the ceftriaxone and penicillin groups were evaluated through the nonparametric Mann–Whitney U-test. Categorical variables were subjected to comparison using chi-squared tests. To mitigate potential treatment allocation bias, all baseline covariates underwent propensity score matching (PSM) before the outcome analysis. Univariate and multivariate logistic regression analyses were performed to identify the clinical response associated with ocular syphilis. All statistical analyses were conducted using R software (Comprehensive R Archive Network Project 4.1.1) and SPSS 26.0 statistical software (IBM, Armonk, NY, USA). A threshold of *P* < 0.05, using a two-tailed test, denoted statistically significant differences.

## RESULTS

### Demographics

**Fig 1:**
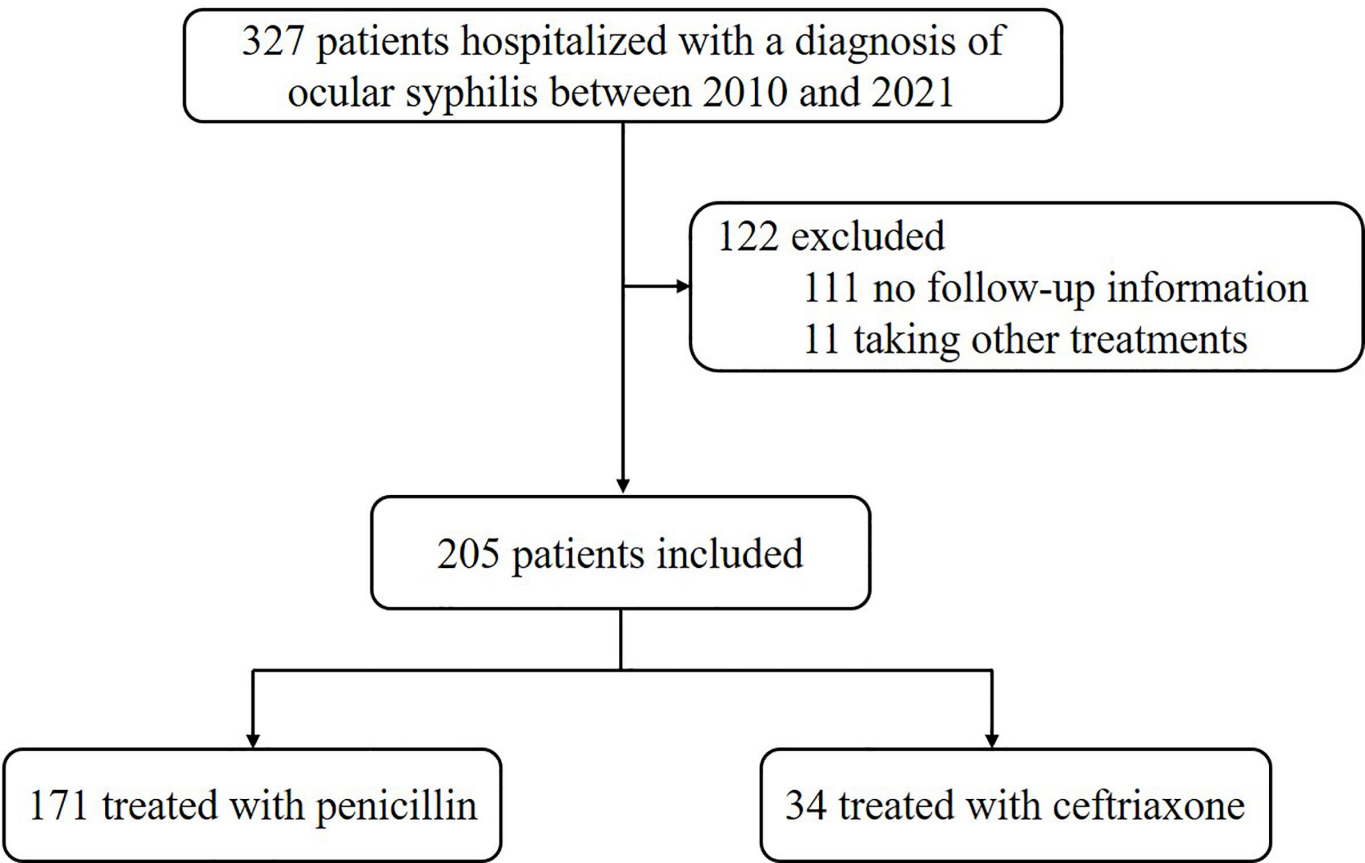
Flow chart showing how patients with ocular syphilis were recruited for analysis.

Between 2010 and 2021, a total of 327 new cases of ocular syphilis were diagnosed at our sexually transmitted diseases (STD) outpatient department. After excluding 111 patients without follow-up data and 11 patients who did not receive penicillin or ceftriaxone treatment, a final cohort of 205 patients with ocular syphilis was included in this study (Fig. 1).

The median age of the patients was 56 years, with an interquartile range of 49–62. The majority of patients were male, accounting for 66.8% of cases (*n* = 137). Fourteen patients were homosexual. Seven patients (3.4%) were HIV-positive. All of them had received antiretroviral therapy, and the viral loads of all HIV patients were lower than the lower detection limit. Additionally, six patients exhibited findings consistent with syphilitic rash of secondary syphilis.

The most common ocular diagnoses were uveitis, representing 48.3% (*n* = 99) of cases. Other subtypes of ocular syphilis included 30 cases (14.6%) of optic neuritis, 16 cases (7.8%) of optic atrophy, three cases (1.5%) of conjunctivitis, and 57 cases (27.8%) involving various other conditions. Unilateral effects were observed in 73 patients (35.6%), while bilateral effects were seen in 132 patients (64.4%).

In terms of serological findings, 72.7% (*n* = 149) of patients had an RPR titer ≥1:32. A positive CSF-VDRL test was found in 58.5% (*n* = 120) of patients. Among the patients, 17.6% (*n* = 36) had a negative CSF-VDRL test but a positive CSF-TPPA test along with elevated total protein levels (>45 mg/dL) or WBC counts (≥8/lL). Furthermore, 23.9% (*n* = 49) of patients had a negative CSF-VDRL test accompanied by normal CSF total protein levels and WBC counts. Bacterial and fungal cultures of CSF yielded negative results for all patients (Table 1; Table S1).

**TABLE 1:** Baseline characteristics of patients with ocular syphilis^*b*^^,*c*^

	Overall
Patients, n	205
Age (years), n (%)	56 (49–62)
Sex, n (%)	
Male	137 (66.8)
Female	68 (33.2)
Coinfection with HIV, n (%)	7 (3.4)
Ocular diagnosis, n (%)	
Uveitis	99 (48.3)
Anterior uveitis	13 (6.3)
Posterior uveitis	52 (25.4)
Panuveitis	34 (16.6)
Optic neuritis	30 (14.6)
Optic atrophy	16 (7.8)
Conjunctivitis	3 (1.5)
Others^*a*^	57 (27.8)
Affected eye, n (%)	
Unilateral	73 (35.6)
Bilateral	132 (64.4)
Serum RPR titer, n (%)	32 (16–64)
≤1:8	29 (14.1)
1:16	27 (13.2)
1:32	48 (23.4)
1:64	51 (24.9)
≥1:128	50 (24.4)
Neurosyphilis, n (%)	120 (58.5)
Presumptive neurosyphilis, n (%)	36 (17.6)
Non-neurosyphilis, n (%)	49 (23.9)

^*a*^ Patients with cranial neuropathy, ocular palsy, or pupil change.

^*b*^ Data are presented as n (%).

^*c*^ HIV, human immunodeficiency virus; RPR, rapid plasma reagin.

The treatment option was decided by the clinicians responsible for patient care. Most of the patients treated with ceftriaxone were allergic to penicillin. Out of the total 205 patients, 171 (83.4%) received treatment with penicillin, while 34 (16.6%) were treated with ceftriaxone. There were no significant differences between the two treatment groups in terms of age, gender, HIV status, ocular diagnosis, affected eye, serum RPR titer, or CSF testsresults (Table 2).

**TABLE 2:** Baseline characteristics of patients according to different treatment regimens^*b*^^,*c*^

	Benzylpenicillin	Ceftriaxone	*P*-value
Patients, n	171	34	
Age (years), n (%)	56 (49–63)	56 (46–62)	0.369
Sex, n (%)			0.912
Male	114 (66.7)	23 (67.6)	
Female	57 (33.3)	11 (32.4)	
Coinfection with HIV, n (%)	5 (2.9)	2 (5.9)	0.328
Ocular diagnosis, n (%)			0.282
Uveitis	85 (49.7)	14 (41.2)	
Anterior uveitis	9 (5.3)	4 (11.8)	
Posterior uveitis	47 (27.5)	5 (14.7)	
Panuveitis	29 (17.0)	5 (14.7)	
Optic neuritis	26 (15.2)	4 (11.8)	
Optic atrophy	14 (8.2)	2 (5.9)	
Conjunctivitis	3 (1.8)	0 (0.0)	
Others^*a*^	43 (25.1)	14 (41.2)	
Affected eye, n (%)			0.120
Unilateral	65 (38.0)	8 (23.5)	
Bilateral	106 (62.0)	26 (76.5)	
Serum RPR titer, n (%)	32 (16–64)	64 (16–128)	0.395
≤1:8	23 (13.5)	6 (17.6)	
1:16	24 (14.0)	3 (8.8)	
1:32	41 (24.0)	7 (20.6)	
1:64	46 (26.9)	5 (14.7)	
≥1:128	37 (21.6)	13 (38.3)	
Neurosyphilis, n (%)	102 (59.6)	18 (52.9)	0.568
Presumptive neurosyphilis, n (%)	30 (17.5)	6 (17.6)	0.988
Non-neurosyphilis, n (%)	39 (22.8)	10 (29.4)	0.410

^*a*^ Patients with cranial neuropathy, ocular palsy, or pupil change.

^*b*^ Data are presented as n (%) or median (interquartile range).

^*c*^ HIV, human immunodeficiency virus; RPR, rapid plasma reagin.

### Baseline covariates after propensity score matching (PSM)

Propensity score-matched cohort analysis was used to balance baseline covariates between the penicillin group and the ceftriaxone group. The baseline characteristics of the post-matched cohorts are presented in Table S2. In total, 136 patients from the penicillin group were successfully matched with 34 patients from the ceftriaxone group. The matching procedure effectively mitigated some of the discrepancies that existed between the two groups prior to matching (Fig. S1 and S2). Notably, we present pre-matched data in our subsequent analysis, as the two pre-matched cohorts exhibited no significant differences in any aspect.

### A comparison of the therapeutic effects of penicillin and ceftriaxone

Effectiveness was defined as meeting all of the following criteria within the 3–6-month post-treatment period: (1) either a negative RPR outcome or a ≥ 4 fold reduction in the titer in blood; (2) either a ≥ 2 fold decrease in the VDRL titer or a return to normal WBC count if previously elevated in CSF; (3) improvement in best-corrected visual acuity by at least one line on the chart. In the penicillin group, treatment exhibited effectiveness in 48.0% of patients (*n* = 82), whereas in the ceftriaxone group, this effectiveness was observed in 38.2% of patients (*n* = 13). The crude odds ratio was 0.672 (95% CI: 0.316–1.428, *P* = 0.301), indicating no significant differences in effectiveness between both groups. Only serological or CSF responses were observed in 28.1% of patients (*n* = 48) in the penicillin group and 32.4% of patients (*n* = 11) in the ceftriaxone group (*P* = 0.615). Only visual improvement was observed in 6.4% of patients (*n* = 11) in the penicillin group, compared to 11.8% of patients (*n* = 4) in the ceftriaxone group (*P* = 0.283). Conversely, treatment exhibited ineffectiveness in 17.5% (*n* = 30) of patients in the penicillin group and in 17.6% (*n* = 6) of patients in the ceftriaxone group (*P* = 0.988) (Table 3).

**TABLE 3:** Treatment effects according to different treatment regimens^*a*^^,*b*^

	Penicillin		Ceftriaxone		*P* value
	n (%)	OR (95% CI)	n (%)	OR (95% CI)	
Effective	82 (48.0)	1 (ref)	13 (38.2)	0.672 (0.316–1.428)	0.301
Improved					
Serological or CSF response	48 (28.1)	1 (ref)	11 (32.4)	1.226 (0.555–2.706)	0.615
Visual improvement	11 (6.4)	1 (ref)	4 (11.8)	1.939 (0.579–6.497)	0.283
Ineffective	30 (17.5)	1 (ref)	6 (17.6)	1.007 (0.383–2.646)	0.988

^*a*^ OR, odds ratio; CI, confidence interval; ref, reference.

^*b*^ CSF, cerebrospinal fluid.

In line with the post-matched data, there were no noteworthy differences in significance between the penicillin group and the ceftriaxone group with regards to the treatment outcomes of ocular syphilis (Table 3; Table S3).

**Fig 2:**
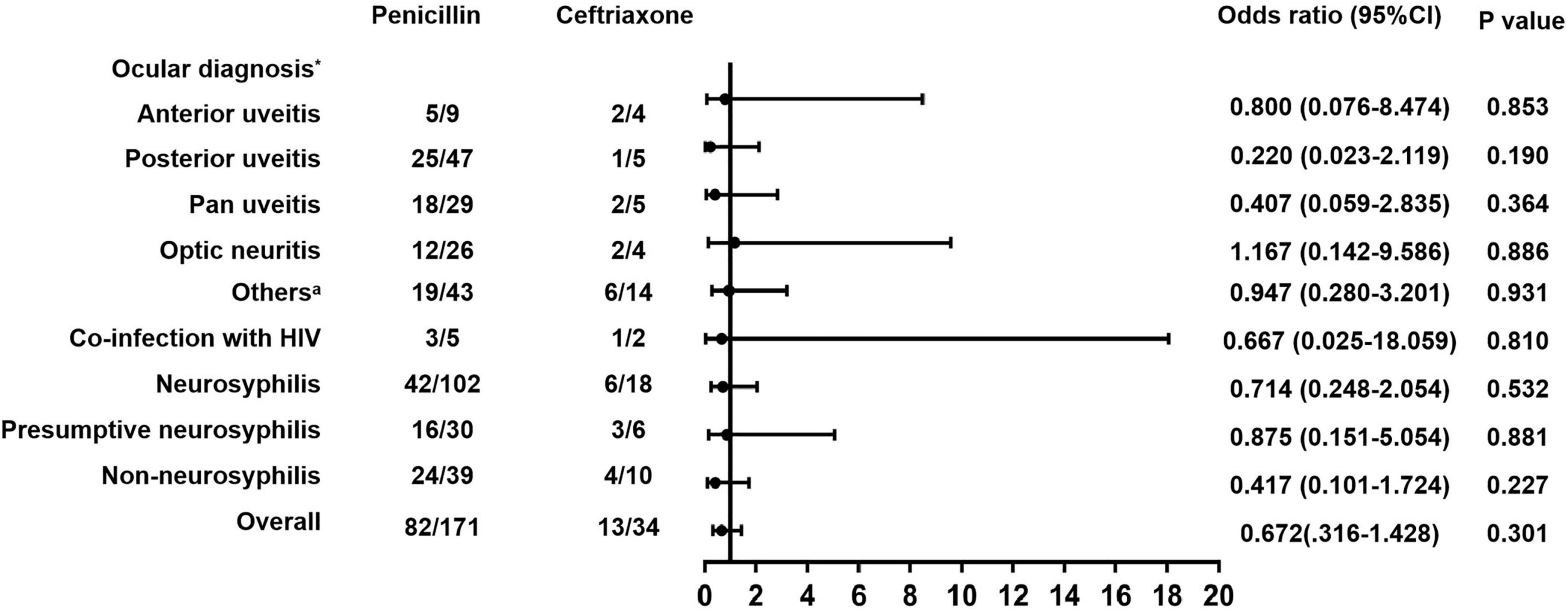
The relative effectiveness of inpatients treated with ceftriaxone or penicillin, based on ocular diagnosis, HIV status, and CSF analysis. * Types of patients diagnosed with ocular syphilis; ^a^ patients with cranial neuropathy, ocular palsy, or pupil change. Subgroup analyses, segmented according to ocular diagnosis, HIV status, and CSF analysis, revealed no marked disparities between the groups in terms of effectiveness.

Moreover, there were no reports of severe adverse effects within either treatment group. Subgroup analyses, segmented according to ocular diagnosis, HIV status, and CSF analysis, revealed no marked disparities between the groups in terms of effectiveness. This finding remains consistent with the post-matched data (Fig. 2; Table S4). However, due to the limited sample size and potential grouping bias for cases of optic atrophy and conjunctivitis, it was impossible to compute the odds ratio for effectiveness in these specific subgroups.

## DISCUSSION

In this retrospective cohort study, our findings reveal that ceftriaxone and penicillin exhibit similar effectiveness in treating ocular syphilis, regardless of patient gender, the specific type of ocular diagnosis, or whether the condition occurs with or without concurrent NS.

Guidelines both in the United States and European Union recommend parenterally administered penicillin G for treating all stages of syphilis (19, 20). For the early stages of syphilis, benzathine penicillin treatment boasts of noteworthy advantages, including safety, effectiveness, and a convenient weekly dosing regimen. However, in cases of NS and ocular syphilis, the administration of intravenous aqueous crystalline penicillin G is necessitated every 4 h. Notably, two key factors that hinder the use of aqueous crystalline penicillin G in China are penicillin allergies and the requirement for hospitalization.

Similar to the United States and the European Union guidelines, the syphilis treatment guidelines in China also advocate the use of procaine penicillin for the treatment of neurosyphilis (21). Nevertheless, there has been a scarcity of procaine penicillin in China for several decades. It is clear that the feasibility of treating neurosyphilis with procaine penicillin is limited.

In China, the administration of desensitization therapy is not common for penicillin-allergic patients, and waiting for desensitization therapy is not a feasible option for individuals with ocular syphilis. Of note, patients with ocular syphilis are distinct from those experiencing more severe types of neurosyphilis, such as general paresis and tabes dorsalis, which can considerably disrupt a patient’s daily functioning and ability to work. However, when ocular syphilis does not result in blindness, its impact on a patient’s everyday life and work is relatively minor. Consequently, it becomes challenging for these patients to accept a hospitalization period lasting up to 2 weeks.

Numerous retrospective studies conducted earlier have consistently indicated that both ceftriaxone and penicillin exhibit comparable efficacies for the treatment of early syphilis (22, 23). Moreover, previous investigation carried out in our hospital has suggested that ceftriaxone can be used to treat syphilis in pregnant women and prevent the occurrence of fetal syphilis (24). Additionally, a recent retrospective study reported similar efficacies between ceftriaxone and benzylpenicillin in treating NS (25). However, the application of ceftriaxone in treating ocular syphilis remains sparsely documented, with only a handful of case reports detailing its utilization (16–18, 26). This scarcity underscores the need for further research and exploration in this specific context.

Uveitis is the most prevalent subtype of ocular syphilis, of which posterior uveitis and panuveitis are the most common manifestations (7). Our study identified a total of 99 patients (48.3%) with uveitis, among which 52 were cases of posterior uveitis and 34 were classified as panuveitis. The inconspicuous nature of ocular syphilis manifestations can sometimes lead to underestimation and treatment delays, resulting in severe consequences, even blindness (12, 27). Conversely, swift intervention tends to yield positive prognostic outcomes (28, 29). Notably, Tucker et al. reported an improvement in visual acuity for 97% of patients, either restoring or attaining normal visual acuity following treatment (30). Similarly, another study documented significant visual acuity enhancement in 117 out of 139 eyes after antibiotic therapy (31).

A multicenter retrospective study conducted in France reported recovery rates of 65% and 85% for eyes at 1 month and the final follow-up, respectively (29). In our study, the treatment response rate, encompassing both effective and improved outcomes, reached 82.4% in the ceftriaxone group, closely mirroring the 82.5% rate observed in the penicillin group. This high rate of treatment effectiveness aligns with findings from other studies. Furthermore, our results are consistent with those of previous research, which noted that ocular response rates were not correlated with HIV status, CSF-VDRL levels, or the specific subtype of uveitis (30–32).

Through our present study, we have established the safety and effectiveness of ceftriaxone as a treatment option for ocular syphilis, highlighting several key advantages over penicillin. First, ceftriaxone is an essential alternative for patients allergic to penicillin, without any contraindications to ceftriaxone treatment. As patients are not required to undergo penicillin desensitization, the waiting before treatment is significantly shortened, ultimately mitigating the potential risks associated with delayed intervention. Second, as a third-generation cephalosporin antibiotic, ceftriaxone boasts of the capability to combat various systemic infections caused by susceptible pathogens. Its commendable tissue permeability facilitates broad distribution across different tissues and body fluids, including penetration through the blood–brain barrier into the cerebrospinal fluid (33). Consequently, ceftriaxone holds potential not only in treating uncomplicated ocular syphilis but also in addressing cases of ocular syphilis compounded by cerebrospinal fluid infections. Most importantly, ceftriaxone features an extended half-life, necessitating only one to two daily treatments. This advantageous attribute opens the door to outpatient treatment possibilities for ocular syphilis patients, substantially alleviating the burdens associated with hospitalization (34).

While our study provides valuable insights, it is important to acknowledge and address its limitations. The retrospective nature of this study stands as a notable constraint, as it could potentially introduce bias into the selection of treatments. In this study, penicillin was the preferred treatment for all ocular syphilis patients, with only those allergic to penicillin receiving ceftriaxone. As a result, there is a significant disparity in the number of individuals in the two groups. Despite our efforts to mitigate bias through the use of propensity score matching, it is important to recognize that certain limitations may still persist. For instance, our study was conducted within a single center, potentially restricting the generalizability of our findings to broader populations and diverse healthcare settings. Additionally, it is worth noting that our study participants exclusively comprised Chinese individuals, which might impact the applicability of our results to more heterogeneous populations. Moreover, the proportion of patients coinfected with HIV in our study was lower than that reported rates in other Western countries (31, 32), possibly influencing the representation of this specific subgroup. Furthermore, the evaluation of ocular effectiveness was primarily based on best-corrected visual acuity, omitting other essential ocular examinations, such as the fundus examinations and fundus fluorescein angiography, which could have provided a more comprehensive perspective on ocular health. Acknowledging these limitations is important in interpreting and applying the results of our study in clinical and research contexts.

### Conclusion

Based on our analysis, it is evident that ceftriaxone can serve as an effective primary treatment option for patients with ocular syphilis, comparable to penicillin. In addition to its therapeutic effectiveness, ceftriaxone offers various advantages, including improved convenience, enhanced patient adherence, and a reduction in the substantial medical burden associated with prolonged hospitalization.

Nevertheless, for a more comprehensive and accurate assessment of the effectiveness and safety of ceftriaxone, the next logical step involves conducting a multicenter, prospective, randomized, and controlled trial. Particularly during the period of penicillin shortage (35), such a trial would significantly contribute to advancing our understanding of the potential of ceftriaxone in treating ocular syphilis and its practical feasibility in real-world scenarios.

## SUPPLEMENTAL MATERIAL

Fig. S1 aac.00080-24-s0001.tif Distributional balance of propensity scores.

Fig. S2 aac.00080-24-s0002.jpg Balance plot showing inverse probability treatment weighting.

Supplemental legends aac.00080-24-s0003.docx Legends for supplemental tables and figures.

Supplemental tables aac.00080-24-s0004.docx Tables S1 to S4.

ASM does not own the copyrights to Supplemental Material that may be linked to, or accessed through, an article. The authors have granted ASM a non-exclusive, world-wide license to publish the Supplemental Material files. Please contact the corresponding author directly for reuse.
