# Supplementary figures and images for: Could ceftriaxone be a viable alternative to penicillin for the treatment of ocular syphilis?
Source: Antimicrob Agents Chemother. 2024 May 6;68(6):e00080-24. doi: 10.1128/aac.00080-24 (PMC11620497; doi:10.1128/aac.00080-24)

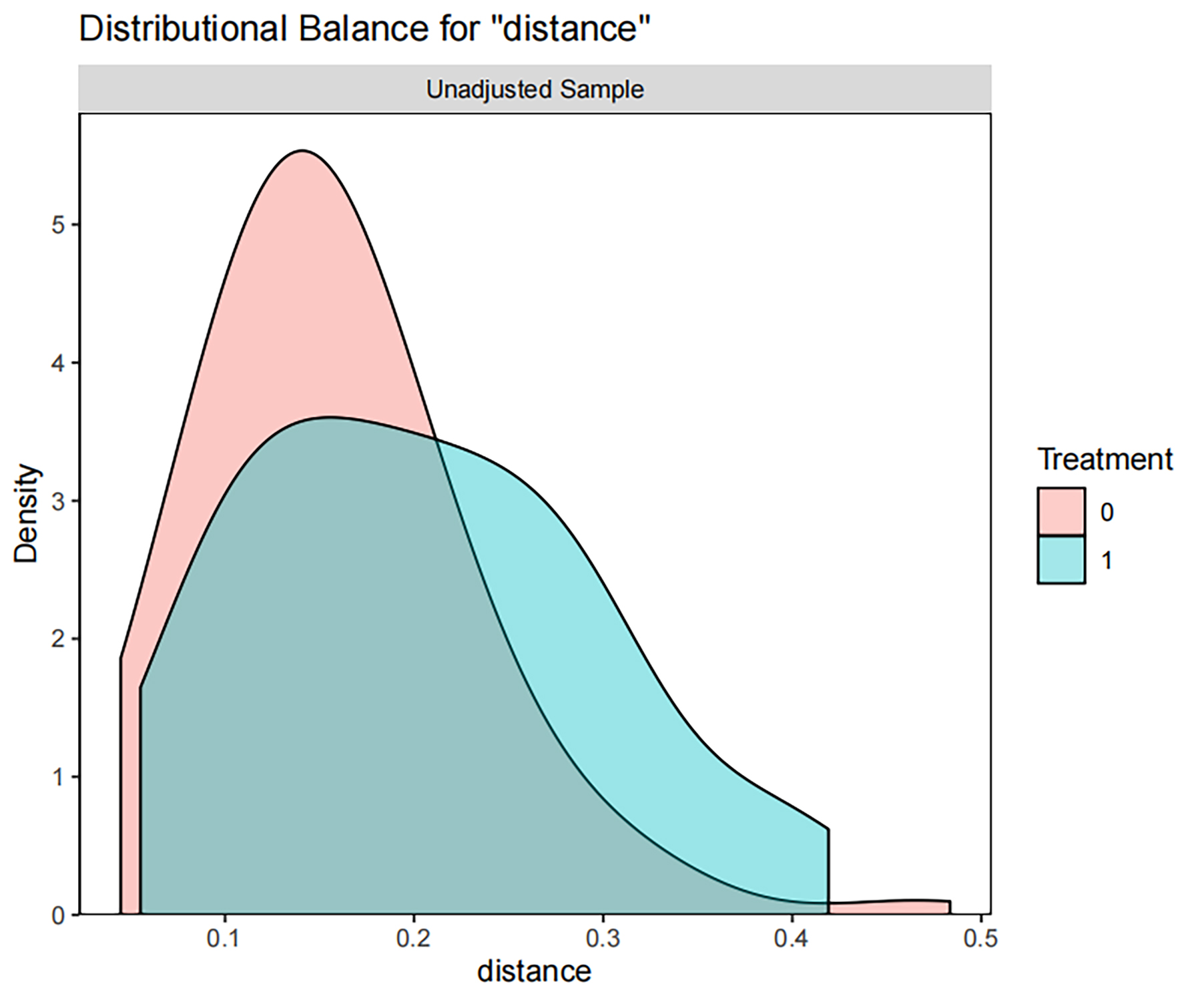

Supplement: Fig. S1 — Distributional balance of propensity scores. [file aac.00080-24-s0001.tif]

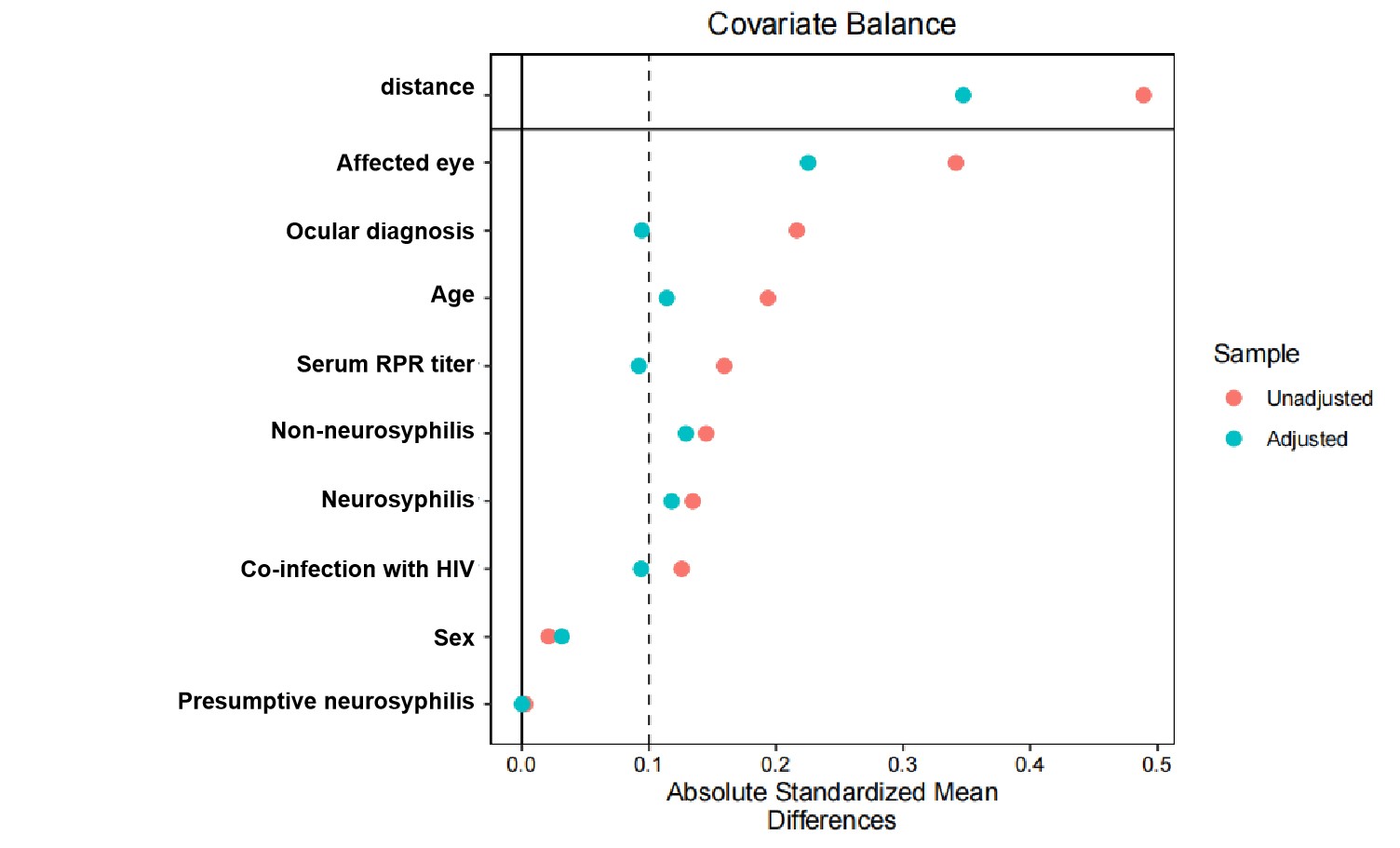

Supplement: Fig. S2 — Balance plot showing inverse probability treatment weighting. [file aac.00080-24-s0002.jpg]
