# Supplementary material for: Could ceftriaxone be a viable alternative to penicillin for the treatment of ocular syphilis?
Source: Antimicrob Agents Chemother. 2024 May 6;68(6):e00080-24. doi: 10.1128/aac.00080-24 (PMC11620497; doi:10.1128/aac.00080-24)
Supplement: Supplemental legends — Legends for supplemental tables and figures. [file aac.00080-24-s0003.docx]

**Supplemental material legends：**

**Table S1.** Characteristics of ocular syphilis patients according to the ocular diagnosis prior to PSM.

**Table S2.** Baseline characteristics of patients according to the treatment regimen after PSM.

**Table S3.** Treatment effect according to different treatment regimens after PSM.

**Table S4.** A comparison of effectiveness rates in patients treated with ceftriaxone or penicillin, based on ocular diagnosis, HIV status and CSF analysis after PSM.

**Figure S1.** Distributional balance of propensity scores.

The two groups exhibited no significant differences.

**Figure S2.** Balance plot showing inverse probability treatment weighting.

The two pre-matched groups exhibited no significant differences in any aspect.
