# Supplementary material for: Could ceftriaxone be a viable alternative to penicillin for the treatment of ocular syphilis?
Source: Antimicrob Agents Chemother. 2024 May 6;68(6):e00080-24. doi: 10.1128/aac.00080-24 (PMC11620497; doi:10.1128/aac.00080-24)
Supplement: Supplemental tables — Tables S1 to S4. [file aac.00080-24-s0004.docx]

| Supplementary Table 1. Characteristics of ocular syphilis patients according to their ocular diagnosis prior to PSM | | | | | | | |
| --- | --- | --- | --- | --- | --- | --- | --- |
|  | Anterior uveitis | Posterior uveitis | Uveitis | Optic neuritis | Optic atrophy | Conjunctivitis | Other ^a^ |
| Age (years), n (%) | 57 (43-64) | 56 (49-63) | 59 (52-63) | 56 (47-58) | 58 (54-64) | 56 (53-58) | 55 (48-634) |
| Male, n (%) | 8 (61.5) | 34 (65.4) | 21 (61.8) | 16 (53.3) | 15 (93.8) | 1 (33.3) | 42 (73.7) |
| Co-infection with HIV, n (%) | 2 (15.4) | 0 (0.0) | 2 (5.9) | 1 (3.3) | 0 (0.0) | 0 (0.0) | 2 (3.5) |
| Affected eye, n (%) |  |  |  |  |  |  |  |
| Unilateral | 7 (53.8) | 26 (50.0) | 9 (26.5) | 12 (40.0) | 4 (25.0) | 1 (33.3) | 14 (24.6) |
| Bilateral | 6 (46.2) | 26 (50.0) | 25 (73.5) | 18 (60.0) | 12 (75.0) | 2 (66.7) | 43 (75.4) |
| Serum RPR titer, n (%) | 32 (16-128) | 64 (32-64) | 64 (32-128) | 32 (16-128) | 64 (32-128) | 32 (8-64) | 32 (16-64) |
| Neurosyphilis, n (%) | 4 (30.8) | 25 (48.1) | 24 (70.6) | 13 (43.3) | 16 (100.0) | 0 (0.0) | 38 (66.7) |
| Presumptive neurosyphilis, n (%) | 3 (23.1) | 11 (21.2) | 7 (20.6) | 7 (23.3) | 0 (0.0) | 0 (0.0) | 8 (14.0) |
| Non-neurosyphilis, n (%) | 6 (46.2) | 16 (30.8) | 3 (8.8) | 10 (33.3) | 0 (0.0) | 3 (100.0) | 11 (19.3) |
| Data are presented as n (%) or median (interquartile range)  ^a^, patients with cranial neuropathy, ocular palsy or pupil change  PSM, propensity score matching; HIV, human immunodeficiency virus; RPR, rapid plasma reagin. | | | | | | | |

| Supplementary Table 2. Baseline characteristics of patients according to treatment regimen after PSM | | | |
| --- | --- | --- | --- |
|  | Penicillin | Ceftriaxone | P-value |
| Patients, n | 136 | 34 |  |
| Age (years), n (%) | 56 (48-62) | 56 (46-62) | 0.625 |
| Sex, n (%) |  |  | 0.871 |
| Male | 90 (66.2) | 23 (67.6) |  |
| Female | 46 (33.8) | 11 (32.4) |  |
| Co-infection with HIV, n (%) | 5 (3.7) | 2 (5.9) | 0.628 |
| Ocular syphilis subtype, n (%) |  |  | 0.533 |
| Uveitis | 58 (42.6) | 14 (41.2) |  |
| Anterior uveitis | 7 (5.1) | 4 (11.8) |  |
| Posterior uveitis | 31 (22.8) | 5 (14.7) |  |
| Pan uveitis | 20 (14.7) | 5 (14.7) |  |
| Optic neuritis | 22 (16.2) | 4 (11.8) |  |
| Optic atrophy | 12 (8.8) | 2 (5.9) |  |
| Conjunctivitis | 3 (2.2) | 0 (0.0) |  |
| Others ^a^ | 41 (30.1) | 14 (41.2) |  |
| Affected eye, n (%) |  |  | 0.310 |
| Unilateral | 48 (35.3) | 8 (23.5) |  |
| Bilateral | 88 (64.7) | 26 (76.5) |  |
| Serum RPR titer, n (%) | 64 (16-64) | 64 (16-128) | 0.514 |
| ≤1:8 | 20 (14.7) | 6 (17.6) |  |
| 1:16 | 17 (12.5) | 3 (8.8) |  |
| 1:32 | 30 (22.1) | 7 (20.6) |  |
| 1:64 | 38 (27.9) | 5 (14.7) |  |
| ≥1:128 | 31 (22.8) | 13 (38.3) |  |
| Neurosyphilis, n (%) | 80 (58.8) | 18 (52.9) | 0.565 |
| Presumptive neurosyphilis, n (%) | 24 (17.6) | 6 (17.6) | 1.000 |
| Non-neurosyphilis, n (%) | 32 (23.5) | 10 (29.4) | 0.508 |
| Data are presented as n (%) or median (interquartile range).  ^a^, patients with cranial neuropathy, ocular palsy or pupil change  HIV, human immunodeficiency virus; RPR, rapid plasma regain. | | | |

| Supplementary Table 3. Treatment effects according to different treatment regimens after PSM | | | | | | |
| --- | --- | --- | --- | --- | --- | --- |
|  | Penicillin | |  | Ceftriaxone | | P value |
|  | n (%) | OR (95%CI) |  | n (%) | OR (95%CI) |  |
| Effective | 62 (45.6) | 1 (ref) |  | 13 (38.2) | 0.739 (0.342-1.595) | 0.441 |
| Improved |  |  |  |  |  |  |
| Serological or CSF response | 37 (27.2) | 1 (ref) |  | 11 (32.4) | 1.280 (0.568-2.881) | 0.552 |
| Visual improvement | 11 (8.1) | 1 (ref) |  | 4 (11.8) | 1.515 (0.451-5.090) | 0.502 |
| Ineffective | 26 (19.1) | 1 (ref) |  | 6 (17.6) | 0.907 (0.340-2.415) | 0.844 |
| OR odds ratio; CI：confidence interval; Ref：Reference  PSM, propensity score matching | | | | | | |

| Supplementary Table 4. Comparison of effective rates in patients treated with ceftriaxone or penicillin, based on ocular diagnosis, HIV status and CSF analysis after PSM | | | | | | |
| --- | --- | --- | --- | --- | --- | --- |
|  | Penicillin | |  | Ceftriaxone | | P value |
|  |  | OR (95%CI) |  |  | OR (95%CI) |  |
| Ocular diagnosis |  |  |  |  |  |  |
| Anterior uveitis | 5/7 | 1 (ref) |  | 2/4 | 0.400 (0.031-5.151) | 0.482 |
| Posterior uveitis | 16/32 | 1 (ref) |  | 1/5 | 0.234 (0.023-2.342) | 0.217 |
| Pan uveitis | 11/20 | 1 (ref) |  | 2/5 | 0.545 (0.074-4.008) | 0.551 |
| Optic neuritis | 9/22 | 1 (ref) |  | 2/4 | 1.444 (0.171-12.232) | 0.736 |
| Optic atrophy^*^ | 2/12 | - |  | 0/2 | - | - |
| Conjunctivitis^*^ | 1/3 | - |  | 0/0 | - | - |
| Others | 18/41 | 1 (ref) |  | 6/14 | 0.958 (0.281-3.263) | 0.946 |
| Co-infection with HIV | 3/5 | 1 (ref) |  | 1/2 | 0.667 (0.025-18.059) | 0.810 |
| Neurosyphilis | 31/80 | 1 (ref) |  | 6/18 | 0.790 (0.269-2.323) | 0.669 |
| Presumptive neurosyphilis | 13/24 | 1 (ref) |  | 3/6 | 0.846 (0.141-5.070) | 0.855 |
| Non-neurosyphilis | 18/32 | 1 (ref) |  | 4/10 | 0.519 (0.122-2.200) | 0.373 |
| ^*^ due to the small size and grouping bias, no statistical calculation was carried out.  OR odds ratio; CI：confidence interval; Ref：Reference  HIV, human immunodeficiency virus; CSF, cerebrospinal fluid; PSM, propensity score matching. | | | | | | |
